# Mr. Cuming, in Reply to Mr. Wilkinson

**Published:** 1805-04-01

**Authors:** Ralph Cuming

**Affiliations:** Romsey


### *To the Editors of the Medical and Physical Journal.*

GENTLEMEN,

IMPRESSED with a due sense of your liberality, I have to request your permission for the insertion of this paper, in reply to Mr. Wilkinson's animadversions, so profusely bestowed upon me in your last Number, and so replete with *urbanity* and *good manners*. In endeavouring to vindicate myself from unmerited and unjust aspersions, I shall not fight the gentleman with his own weapons, which are empoisoned with invective and abuse, but will endeavour to keep myself within the sphere of moderation, from respect to myself and your publication, whose pages I should be unwilling to pollute by a spirit of recrimination, dictating, offensive, and unbecoming language; and will appeal, as I advance in my present pursuit, to such of the profession as are lovers of science, and desirous of improving the art of medicine, for their opinion, whether Mr. W's remarks are not likely to obstruct the path of medical knowledge, by deterring his professional brethren from publishing their observations, for fear of being arraigned at the tribunal of his *unmerciful*, or some other person's Cynical, criticism; because their communications may not be couched in such terms as may hold accordance with the delicate sympathies of their highly refined conceptions.

Mr. W. begins, by commending my zeal and industry, in pointing out such modes of treatment as I have found successful in practice; but blames me for what he stiles a confident, positive, and assuming manner of enforcing them. As a specimen of my manner, I beg leave to adduce the following quotation from my paper in No. 58, p. 497, when speaking of the efficacy of zincum vitriol. in the cure of agues.

"I only ask some of my medical brethren, such as are not acquainted with its virtues, (for there are certainly many who never heard or thought of its powerful effects) to give it a fair trial, whenever opportunity may serve."

Is this assuming? It is true I appeal with *confidence*, well knowing from the result of my own experience, that such gentlemen as are not wrapped up in self sufficiency, will find their expectations founded on my representation realized. Does Mr. W. wish to have truths told in a wavering, equivocal manner? To adopt such a plan, would

 ill ill accord with the views of every benevolent man, in promoting the advancement of medical knowledge. It really affords me great comfort, and cause for exultation, that the soundness of my doctrine, and validity of my positions, have been lately evinced by a gentleman, who in all probability may not be inferior to Mr. W. either in point of discernment, or professional acquirements; I allude to Mr. Cooke, who in page 527, of No. 70, corroborates my assertions, by a statement of two cases, wherein the zinc. vitriol. effected a cure, when in the first instance, the cortex, and solutio arsenici, had failed.

Mr. W. also accuses me of impatience of contradiction, and want of temper; indeed, it would appear that his own temper and disposition are none of the best. I would ask him, what right he has to interfere with Dr. Scott and myself? The Doctor surely did not ask him to enter the field as his champion; then what could be his motive? It surely was not to excite fresh discord, and disseminate contention, as the matter of his paper has this tendency. I would again demand of him, "by what authority he doeth these things?" and whether he is another "Gallio, who careth not for these things?" He cavils with the spirit of a true Cynic, at my having mentioned in my paper of No. 58, that the remarks I had then made, were the result of *unerring observation and experience.* His ill-natured remarks on this passage, will not effect its conversion into ridicule and contempt, which seems to be his aim; for he remarks, "these are Mr. C's own words," and takes care to have them printed in italics. My observation, I conceive, might justly be said to be unerring, if its truth was proved by the test of experience; then let me appeal to my readers, if his outré comment can be considered consistent with that dignity of sentiment, and decorum of conduct, which should influence the mind of a gentleman.

Mr. W. observes, that I display a want of temper to *those* who differ in opinion with me; I reply to this assertion, that it is not correct nor liberal, for him to speak in the plural, when Dr. S. was the only person who differed with me.

Mr. W. says, in my paper on Leprosy, in No. 69, that I treat the flour bag of the late Dr. Cullen in a disrespectful manner. I should be glad if his remark was entirely confined to the *bag*; but it appears that he suspects the character of the Doctor to be implicated in this dusty discussion which, if my conjecture is right, his supererogation of zeal, inin defence of the venerable and worthy preceptor, has hurried him out of the track of reason; and it behoves me to repel this, and all his unfounded and self-fabricated stories. I defy him to point out any passage in my paper wherein I made use of these, *his own* words, the "flour-bag of the late Dr. Cullen," which he wishes to father on me; indeed, it appears very evident, that there is a great want of candour and charity in this Mr. W's critique, for he declares, that it is "so called by Mr. Cuming, No. 69." Pray have the goodness to observe his note marked thus || in No. 72, and then do me the favor to turn to No. 69, when I am persuaded you will think with me, that any man daring to coin whole sentences, for the sake of injuring a person's feelings, or loading him with unmerited censure, should be held up to public contempt. Can such conduct be considered as gentlemanly? He now states, alluding to my paper on leprosy, No. 69, "I shall not enter into a discussion of this case, which he after having, as he says, cured as a general affection, considered it as local, from its continuing in the form of fissures, &c. in the palms of his hands; and which he says, gave way to the application of a blister." In this instance he gives me another opportunity of exposing him to the censure of every honest man; the words of the above quotation are his own, and not mine. It is easy to vilify any person, provided the undertaker of this benevolent office should condescend to coin words, and assert in the most bare faced manner, that they were those of his adversary. I intreat my reader to consult my paper, and observe, whether I have said, that after I cured a *general* affection I considered it as *local*. This Mr. W. must have forged, for the sake of exposing me to ridicule and derision; but instead of a laugh intended at my expence, a contemptuous sneer will be retorted on him for such unmanly arts. Vox veritatis ad cælum volat. I must candidly confess I feel a strong propensity to treat Mr. W. in the way which his conduct so richly merits; but "bear, and forbear," shall have a becoming controul over my pen.

I observed in the paper which Mr. W. has so mangled, misquoted, and added to, "that though the cutis vera is the seat of the disease, it may be considered as partaking more of the nature of a local than a general affection." For who ever considered the itch as a disease of the habit? yet it pervades the whole body.

He now begins to praise me, and gives me credit for

 ingenuity; ingenuity; on which account I must thank him, bearing in mind the good old adage, "Date Cæsari qua sunt Cæsarea."* A little further on, he observes, "yet the propensity or itch of snarling at our old masters, for it appears that Mr. C. from his own confession was a disciple of this great man," alluding to Dr. Cullen; I must again contradict Mr. W. whose erroneous (to say nothing worse of them) assertions, are so truly unaccountable; however proud I might be, of having had so great a preceptor, I must disclaim all pretensions to this honor, and challenge my adversary to point out, in any part of my writings, wherein "*I confess*" myself, as having been this pupil of Dr. C. He surely does not wish to impeach me of ingratitude! I shall tell him whose pupil I was; in 1792 and 1793, I attended the class of that amiable man Dr. Gregory, where I think, if my memory does not fail me, that I have seen Mr. W. who was pointed out to me as a man of ingenuity, for having invented a machine for the inflation of the lungs.

Having held up to public view, some of Mr. W's errors, I shall content myself with skimming over the remainder of his paper, in a cursory way. He accuses me of "barking at the dead old lion, and from whose *prey* himself had been nurtured when a young helpless cub." As I do not understand the sense of this "*and from whose prey*," I shall be silent; though it may be very clever, it is beyond my conception. I have heard of whelps barking, and bears growling when they had got a sore head; but as I never hurt either Mr. W's head or heart, I do not know why he has attacked me.

Mr. W. again observes, "if I have noticed this error, or rather mark of disrespect, of Mr. C. to the memory of his late admirable preceptor, Dr. Cullen." I demand of him to prove to any liberal minded man, that I have been at all disrespectful; on the contrary, I can prove this assertion does not rest upon the basis of truth, by a quotation from my paper, No. 69, p. 406. "During my studies at a celebrated medical school, the flour-bag was the only application then in vogue for erysipelas; from an idea, that it was dangerous to employ refrigerants; medicines of this class are frequently termed repellents; and it would have been a happy circumstance for many patients, if their medical

---

* I give Mr. W. as *Cæsar*, my thanks.

medical attendants had not been frightened out of their wits, at the thought of this terrible name *repellent!"* The flour-bag which Mr. C. has so artfully tacked to Dr. Cullen, you perceive, is not mentioned by me, in conjunction with the venerable name of Cullen. This part of my paper relates to the practice which obtained in the Edinburgh Infirmary, the school I alluded to; and which the generality of students attend.

Now, for my disrespect to Dr. Cullen, these are my words: The famous Cullen, when treating on erysipelas, seems to conceive that no other application, save meal or flour, is admissable, except when there is a sense of throbbing in the part, indicating suppuration; yet I must dissent from such an opinion, though sanctioned by so great a writer. Is this the language of disrespect, the *famous* Cullen! so *great* a writer! Is it possible to be more respectful? But if Mr. W. considers dissention in opinion disrespectful, and this idea should become prevalent, we may bid adieu to every improvement in the profession. Thus it will appear, that Mr. W. has, Don Quixote like, been combating a phantom, which existed no where, save in his own brain; and if he hugs himself with the thoughts of having repelled "certain disrespectful insinuations aimed lately by a gentleman (with whom, speaking of himself, I differed in opinion) at the professional character of the late ingenious and benevolent Dr. Fothergill," I have a strong presentiment that this, his last attempt on me, will neither redound to his honour, nor give him cause for exultation; unless he has discovered a method of repelling a non-entity. He now takes his leave of the illustrious Cullen, and takes up the cudgels for one who, when put in competition with the other, may only be considered as a satellite; yet, strange to tell, his language, which before was only tinctured, becomes now surcharged with scurrility. "Mr. Cuming's disrespectful, and I fear, intemperate reply to Dr. Scott, more particularly as the Doctor, from the *ungentleman-like manner* in which he appears to have been treated," I shall be glad if my reader will have the condescension to read my reply to Dr. Scott, in No. 61, vol. 11, where I am confident he will find nothing either harsh or unbecoming. Let me now ask, if Mr. W. has acted the part of a gentleman towards me, in thus (without the smallest provocation) loading me with the opprobrious epithet *ungentleman-like?* indeed, it appears evident from my refutation of all he has hitherto advanced, that he has not not a proper regard for truth, and that he conceives himself at liberty to say any thing he pleases. In another place he says, "I advance not these remarks from ill temper, enmity, or disrespect to Mr. Cuming." Can he consider such language complimentary! In a word, if this Mr. Wilkinson's knowledge of men, manners, and his duty as a member of civilized society, be so confined, he rather, than resentment, merits pity! For an answer to the whole of his critique, on my reply to Dr. Scott, I refer my readers to that paper; and assure Mr. W. that I cannot find the preparation of cuprum ammoniacum in that edition of the Edinburgh Dispensatory in my possession, printed in the year 1789. I have, since the perusal of Mr. W's *philippic,* perused the work with attention, and do still aver, that I cannot find it; alluding to the *preparation,* it is not mentioned in the index, nor is there a recipe for it; it is true, I now perceive, that under the head of copper, the name is mentioned; and it would appear, that they are apprehensive of the ill effects which might accrue from its use; otherwise, why is not the recipe found. With respect to his observations on my plan of treating sphacelus with nitre, they appear very frivolous; if he has stored up in his fund of knowledge a better remedy, the sooner he divulges it the better. He remarks, "should it hereafter appear that both these easily procurable remedies prove equally beneficial in the hands of others, his claim to *originality* in these will scarcely be disputed." I have, since these quaint remarks of his were written, witnessed the happy effects resulting from my practice, in a most deplorable case, which I published in No. 70, vol. 12; when I requested as a *particular favour,* that some of my medical brethren would, when opportunity offered, publish the result of cases which might occur in their practice; shonld they be disposed to place that confidence in it, which its efficacy so justly entitles it to. Mr. W. observes, speaking of zincum vitriol. which I recommended for the cure of agues, that I said, "it deserves the name of specific;" and that I never knew it fail. Never knew it fail is true; but, to say nothing worse of the former part of his sentence, it is extremely erroneous, and a declaration without foundation; for my words in No. 58 are, *more* entitled to the epithet specific. I appeal to common sense, if Mr. W. does not wantonly pervert whole sentences, for the sake of gratifying his *benevolent disposition.*

He finally concludes, that his remarks are given fairly and and candidly; whether this is the case or not, I have great pleasure in leaving to the decision of such men as will neither add nor diminish, nor condescend to fabricate aught, for the sake of unjust crimination.

I shall now take my conjé of Mr. Wilkinson, assuring him, that neither motives of enmity nor revenge have dictated my reply: I know, that my tenets are not taken from that sect who hold it just to retaliate, agreeably to the old law *of tooth for a tooth, and eye for an eye.* No, far different considerations influenced my mind; a manly sense of the injustice done me will sufficiently apologize, were any apology requisite, for repelling unmerited insult and unjust aggression; and, whether I have succeeded in my endeavour, will be best known by those who are not parties in the affair. I freely forgive Mr. W. and if he is not contented that the business should end here, let him appeal, as I have done, to the candid and unprejudiced, by whose decision I shall abide; hoping that the case at issue between us will appear as evident as that between Dr. Trotter and Dr. Clarke, with these exceptions, that the subject matter in debate is different; and that some people may suppose it necessary, audire alteram partem, whose silence does not admit of the most favourable construction; but Mr. Wilkinson does me the honour to insinuate, that some importance may be due to my communications.

I am, &c.

*Romsey, Feb.* 12, 1805. RALPH CUMING.

